# Factors affecting trust in Bangladesh police among urban male residents: a test on instrumental and expressive model

**DOI:** 10.3389/fsoc.2025.1480164

**Published:** 2025-02-27

**Authors:** Md Khalid Hasan, Maruf Hasan Rumi, Abu Hossain Muhammad Ahsan

**Affiliations:** ^1^Institute of Disaster Management and Vulnerability Studies, University of Dhaka, Dhaka, Bangladesh; ^2^Department of Public Administration, University of Dhaka, Dhaka, Bangladesh

**Keywords:** trust, urban men, instrumental model, expressive model, Bangladesh police

## Abstract

**Objectives:**

The primary aim of this study was to examine the level of trust male residents in urban areas of Bangladesh have in the police. Additionally, the study sought to explore the various factors that influence this trust.

**Method:**

This study employed a robust cross-sectional research design. Utilizing multi-stage sampling techniques, the survey was conducted among male respondents (aged 18 or over) through in-person interviews, and the data was collected using the Kobo Toolbox. A total of 1,108 data were collected from four city corporation areas in Dhaka, Sylhet, Khulna, and Rangpur districts, ensuring a comprehensive representation of urban areas in Bangladesh.

**Results:**

The study revealed that male citizens in urban areas of Bangladesh have a moderate level of trust in the police. Trust in neighbors was found to be a significant and positive factor influencing trust in police. The study also found that neighborhood relative safety was positively associated with trust in police, while the perceived crime problem in the locality had a negative impact on trust in police. Furthermore, middle-aged (39–49 years old) and older men (over 50 years old) were found to have significantly lower trust in police than young adult (19–39 years old) respondents. Location was also a crucial factor, with respondents from Khulna City exhibiting substantially higher trust in the police than male residents of other cities.

**Conclusion:**

The study underscores the need for the law enforcement agency to address the current situation. To improve public trust in the police, it is crucial that the agency increases its social media presence and launches campaigns to highlight its recent successes.

## 1 Introduction

The pattern of crime and criminal activities has been drastically changed due to globalization and rapid urbanization in the developing world (Rahman, 2013). In their urban centers, crimes are exacerbated by poverty, inequality, higher population growth and density, and poor urban planning, design, and management (Arias, 2011). In this given context, the task of crime prevention and law enforcement became significantly challenging for police. Hence, establishing interaction and trust between police and citizens has become even more crucial. Citizen’s response, assistance, and collaboration with the police are contingent upon their perception and evaluation of the police performance (Wu and Sun, 2009). Without public support, police often fail to achieve their goals of ensuring public safety (Cao et al., 1998). However, there are several conceptual distinctions in measuring citizen’s evaluation and assistance to the police force, for instance, trust, confidence, and satisfaction with police (Frimpong et al., 2019). Scholars defined these terms in different ways, such as trust in police is connected to the performance of police assessment of police in an institutional setting, Trust in the police is correlated with the police officer’s interpersonal relationship with the citizens, and satisfaction in the police is derived from the service delivery and quality of police actions to reduce crime (Bradford and Jackson, 2010; Lowe and Innes, 2012; Stanko et al., 2012; Frimpong et al., 2019). Despite conceptual distinctions, those measures are interconnected, and the flexibility was utilized to cover the dearth of research on citizens’ trust in police.

Nevertheless, trust in police is a multifaceted concept affected by socioeconomic, historical, cultural, and situational variables (Weitzer and Tuch, 2005; Ivković, 2008; Alalehto and Larsson, 2016). For example, Wu and Sun (2009) observed trust variations among groups in China based on sociodemographic characteristics like gender, age, education, source of income, and employment position. In the USA, Weitzer and Tuch (2002) explored the implications of race, social class, and prior experience of police in determining citizens’ trust in police. Moreover, Kääriäinen et al. (2016) measured the public trust in response to an incident of police misconduct in Poland based on public observations and images concerning the police force. Thus, the level of trust and its associated factors are not universal across times and regions but rather much more of a sociodemographic and contextual issue. Throughout history, the police have been employed as a tool of the state to suppress dissent and safeguard the interests of colonial forces in the Indian subcontinent. As a result, police were alienated from the masses, and that estrangement brewed severe distrust over the police’s role and sincerity that still resonates in the early part of the twenty-first century (Baixas, 2008; Grant et al., 2022). This presents a unique situation to be explored regarding police reforms and the digitalization of services in a developing country (Uddin, 2009; Islam et al., 2020; Grant et al., 2022).

Notwithstanding contextual factors, demographics, especially gender, have been a significant focus of trust studies concerning police (Hu et al., 2015; Chambers et al., 2020). However, research examining the impact of gender on the police’s perceptions sparked mixed results (Brown and Benedict, 2002; Hurst et al., 2005; Sun et al., 2013b). Gender differences in perception toward police have been found in several studies, and most of them reported that female citizens possessed greater trust than their male counterparts (Lai and Zhao, 2010; Han et al., 2017). However, some researchers argued that a higher level of trust among women corresponds with fewer police encounters, which lowers the possibility of negative experiences (Han et al., 2017; Hitchens, 2020; Edzes, 2021). Han et al. (2017) found that females were treated more favorably during any kind of police procedure. Similarly, Gabbidon et al. (2011) found that when interacting with police officers, women are often treated more equitably than men. However, from a socio-cultural lens, men in households engage in more public activities compared to women due to gender role stereotyping and a masculine culture in developing nations (Terzi et al., 2022), which leads to a higher likelihood of being or experiencing being affected by criminal activity (Denno, 1994). Hence, researchers in developing countries are increasingly concerned with the perception of police performance and the level of trust placed in them from a gendered perspective (Hu et al., 2015; Wu et al., 2016). It is then justifiable to explore men’s trust in police due to their frequent exposure and interaction with police more than their counterparts in developing countries such as Bangladesh. However, limited literature has been found in Bangladesh that explores urban men’s trust in the police. The present study fills the gaps and has the potential to significantly contribute to the understanding of trust in the police force. Thus, this study aimed to assess the level of trust in Bangladesh police among urban males using instrumental and expressive models and examine the factors influencing their trust in police.

## 2 Models for measuring trust in police

Trust is the inherent faith that develops when a person in a specific position consistently performs their responsibilities with integrity and in accordance with societal norms (Hawdon, 2008). Tyler and Huo (2002) measured trust in police from two dimensions: outcome-based trust and procedural-based trust. While the former is associated with the public’s expectation of justice in the police’s decision-making process, the latter is related to the impartial police services provision for the communities (Sunshine and Tyler, 2003). More distinctively, according to procedural concerns, how individuals assess and perceive the fairness of police officers actions, including respect and equal treatment, significantly influences their confidence in the police (Tyler, 2005). Conversely, outcome-based trust stems from the citizen’s assessment of police performance, which is associated with concerns about insecurity and disorder and the effectiveness of crime prevention responsibility of the police force (Jang et al., 2010; Zhorayev, 2020). When citizens form positive impressions of police, they feel safe and secure around them (Jackson et al., 2011).

Numerous models were used to measure and establish empirical relationships between citizens’ perception and trust in police, but in most cases, researchers typically conceptualized trust through instrumental, expressive, and normative models for both developing and developed country contexts (for example, see Bradford and Myhill, 2015; Sun et al., 2013a). Most studies concerning trust models are in the context of developed countries, and very few are in developing countries of the Global South (e.g., Lim and Kwak, 2022; Park et al., 2021; Sun et al., 2014). However, the instrumental model and expressive model have been used extensively by researchers, similar to the current study, to identify factors of trust from a theoretical perspective (Frimpong et al., 2019; Han et al., 2020).

According to the instrumental model, the police’s capacity to reduce crime and instill dread of crime in society is a critical factor in establishing the trust of the public. Firsthand experience of crime and victimization are a few sources of instrumental measures of trust in the police force (Bradford and Myhill, 2015). The instrumental model demonstrates that the police are essential in crime prevention and the cultivation of a sense of security among the people (Sun et al., 2014). When policing fails to meet citizens’ expectations, it is considered ineffective, leading to decreased public confidence in the police. However, the instrumental role of the police is to contribute systematically to police performance (Sindall et al., 2012). This fear of crime victimization negatively impacts the collective social identity and leads to a cyclical process of increased crime and disorder (Alda et al., 2017).

In contrast, the expressive public trust paradigm in the police posits that citizens’ perceptions of the police in the context of neighborhood order, solidarity, and informal social control are influenced by expressive interests (Jackson and Sunshine, 2007; Sun et al., 2013b). In essence, residents desire the police to combat crime and uphold the neighborhood’s decency and values. A police force gains support and credibility by following community standards and values. Thus, public trust is tied to believing that police officers will act in the community’s best interests (Tyler and Huo, 2002). In the expressive model, police are judged symbolically as “agents of reform,” and they will lose public trust when citizens perceive that their neighborhood norms and values are deteriorating (Jackson and Sunshine, 2007); not only the police’s performance in maintaining a crime-free community but also the trust in law enforcement is influenced by the trust in the neighborhood (Han et al., 2020). Research has demonstrated that confidence in law enforcement and the fear of crime are significantly influenced by community cohesion (Alda et al., 2017; Sargeant and Kochel, 2018). Consequently, when they perceive that they live in a good community, police become a part of the neighborhood, demonstrating the desire of individuals to assist the police (Jackson and Bradford, 2009).

Furthermore, citizen’s voluntary cooperation with police and their support of police actions are essential to promote efficacy in the police force (Pryce and Gainey, 2022). Similarly, The public’s impression of the police’s credibility is influenced by their performance, responsibility, and moral alignment with society, while citizens’ contentment with law and order maintenance is strongly correlated with their faith in the police (Wahyurudhanto, 2022). Furthermore, police are respected for upholding social norms and ideals (Jackson and Sunshine, 2007). Moreover, public trust in the police is vital for effective policing, as it encourages individuals to abide by the law and work in tandem with the police. Ineffective crime control or law enforcement is the result of a dearth of public confidence and support for the police. This can have severe political repercussions, including dangers to the legitimacy of the government (Wu et al., 2012).

## 3 Methods

### 3.1 Study design

This study is part of an extensive investigation into the trust of Bangladeshi urban residents in the police conducted by Ahsan et al. (2024). It employed a cross-sectional research design and utilized a quantitative approach. Data were obtained through direct engagement with participants, using a mobile data collection tool and a structured questionnaire, ensuring a comprehensive understanding of the subject.

### 3.2 Sample and settings

Four city corporations of the divisional centers were selected through multi-stage sampling techniques for this study. The study population comprised urban residents from 10 wards (the smallest local government unit in Bangladesh) in each city corporation area. Selection at all stages was based on simple random sampling to ensure an equal chance of selection for all units of analysis. The respondents were 18 years of age or older and had sought assistance from the police within the last 24 months, as it mitigates recall bias and guarantees that responses are based on recent interactions, thereby more accurately representing contemporary policing practices, policies, and service quality, ensuring that a wide range of perspectives were represented. In the end, a total of 1,640 respondent data were collected, including 1,108 from male urban residents.

### 3.3 Instrument and data collection

The survey used Kobo Toolbox, a data collection software to collect data from Dhaka, Sylhet, Khulna, and Rangpur city corporations. The structured questionnaire, consisting of 40 items in five sections, covering variables for instrumental, expressive, and normative models of trust in the police, was employed. The data collection occurred from December 2021 to January 2022. A pilot test was carried out with 25 urban residents to assess the face and criterion validity of the questionnaire. The questionnaire was initially created in English and later translated into Bangla to allow for inclusive data collection from randomly chosen households in each ward.

#### 3.3.1 Dependent variable

Trust in the police, the dependent variable, was appraised using procedural-based and outcome-based trust models. For this purpose, a nine-item scale was developed and validated, with each item evaluated using a 6-point Likert scale response option. A higher score indicated greater levels of trust in the police among respondents. The principal component analysis (PCA) resulted in a unifactorial solution, explaining roughly 70.2% of the total variance. The factor loadings ranged from 0.757 to 0.871. The Kaiser-Meyer-Olkin (KMO) test for sampling adequacy produced a value of 0.956, and Bartlett’s test for sphericity was statistically significant (*p* < 0.001), signifying the suitability of principal component analysis. The scale’s Cronbach’s alpha coefficient was 0.947, illustrating satisfactory internal consistency (Table 1).

**Table 1:** Descriptive statistics and factor loadings of items measuring trust in the police.

Dimension	Items	Factor loadings	Min–Max	Mean	Std. Deviation
Procedural-based trust	1. How sincere and patient are the police while functioning with the citizens?	0.858	0–5	2.65	0.990
	2. To what extent do you believe police statements or comments about any incident?	0.807	0–5	2.72	0.845
	3. Do the police treat people with respect and friendliness while dealing with cases?	0.866	0–5	2.60	1.097
	4. Do the police perform their duty by considering the needs of the local people?	0.866	0–5	2.83	1.025
	5. Do the police take necessary actions to combat crime in your neighborhood?	0.818	0–5	2.94	0.957
	6. In your opinion, how do the police give people enough chance/time to explain their situation?	0.859	0–5	2.50	1.045
Outcome-based trust	7. To what extent do you feel confident in police in this area?	0.871	0–5	2.78	1.016
	8. Do you think the police investigate cases fairly and impartially?	0.833	0–5	2.63	0.938
	9. In case of emergency response, how efficient do you think the police are in your area?	0.757	0–5	2.97	0.921
Extraction Method: Principal Component Analysis					
Eigenvalue = 6.322, Cumulative % of variance = 70.242, Cronbach’s alpha = 0.947					

KMO and Bartlett’s Test		
Kaiser-Meyer-Olkin Measure of Sampling Adequacy		0.956
Bartlett’s Test of Sphericity	Approx. Chi-Square	8,808.03
	df	36
	Sig.	<0.001

#### 3.3.2 Independent and control variables

The independent variables were chosen based on the instrumental and expressive models of public trust in the police. These variables included concern for personal and family safety, victimization history, perceived crime problems in the neighborhoods, personal safety awareness, neighborhood relative safety for the instrumental model, trust in neighbors, and the law-and-order situation for the expressive model.

Additionally, control variables were grouped into four categories: general characteristics, media influence, satisfaction with law and judiciary, and locality. General characteristics covered gender, age, educational attainment, occupation, religion, family composition, and income. Media influence variables included knowledge and belief in media reports about the police. Satisfaction with governmental law and order operations examined participants’ satisfaction regarding crime control measures in the selected area, and the trial of criminal cases by the judiciary investigated their satisfaction with the judicial process of criminal cases, both of which constituted another set of control variables. Lastly, the locality category included geographic regions or districts.

### 3.4 Data analysis

For our research, we utilized IBM Statistical Package for the Social Sciences (SPSS) version 25.0 for Windows (SPSS, Chicago, IL, USA) to conduct both descriptive and inferential statistical analyses. Descriptive statistics were used to report the characteristics of the study variables, including mean, standard deviation, minimum, and maximum values for both dependent and independent variables, as well as control variables. Frequency and percentage were used to report sociodemographic characteristics (Table 2). Furthermore, we calculated mean, standard deviation, f/t statistics, and *p*-values for the participants’ sociodemographic and education-related attributes to assess mean and mean differences of trust in police between or among categories (presented in Table 3).

**Table 2:** Descriptive statistics for variables.

Variables		*n*	%	Mean	Std. Dev	Min	Max
Dependent variable							
	Trust in police			2.74	0.82	0.11	4.78
Independent variables							
Instrumental model	Victimization			0.18	0.38	0	1
	Concern about personal and family safety			2.58	1.02	0	5
	Perceived crime problem in the area			2.61	0.95	0	5
	Personal safety awareness			3.68	0.87	1	5
	Neighborhood relative safety			3.27	0.98	0	5
Expressive model	Trust in neighbors			3.70	0.86	0	5
	Law and order situation			0.59	0.49	0	1
Control Variables							
Socio-demographic	Age (Years)			39.62	11.36	19	75
	19–29	260	23.5				
	30–39	318	28.7				
	40–49	300	27.1				
	≥50	230	20.8				
	Marital status						
	Single	160	14.4				
	Ever married	948	85.6				
	Education						
	No schooling	213	19.2				
	Primary level (I–IX)	379	34.2				
	Secondary level (X–XII)	381	34.4				
	Tertiary level (≥XIII)	135	12.2				
	Primary occupation						
	Service holder	145	13.1				
	Business	321	29.0				
	Student	51	4.6				
	Hawker	67	6.0				
	Labor	137	12.4				
	Farmer	50	4.5				
	Rickshaw puller	117	10.6				
	Auto rickshaw puller	96	8.7				
	Others^a^	124	11.2				
	Religion						
	Islam	977	88.2				
	Hindu	131	11.8				
	Family structure						
	Single	678	61.2				
	Joint	430	38.8				
Media Influence	Believe positive media reports on police			2.77	0.78	0	5
	Believe negative media reports on police			3.21	0.69	1	5
Satisfaction with	Governmental law and order operation			2.68	1.10	0	5
	Trail of criminal cases by the judiciary			2.13	1.27	0	5
Location	Dhaka City	305	27.5				
	Khulna City	276	24.9				
	Rangpur City	219	19.8				
	Sylhet City	308	27.8				

a Includes car diver, fisherman, tailor, handcrafter, aged, unemployed, and householder.

**Table 3:** Mean and mean difference of trust in police between or among categories of the study participants’ socio-demographic and education-related attributes (*n* = 1,108).

Attributes	Trust in police				
	Mean	Std. Deviation	F/t statistics	*p*-values	Post Hoc
Age (Years)^α^			4.810	0.002**	
19–29	2.80	0.80			
30–39	2.80	0.77			
40–49	2.74	0.85			
≥50	2.56	0.87			
Marital status^ǂ^			−1.578	0.115	
Single	2.64	0.72			
Ever married	2.75	0.84			
Education^α^			1.925	0.124	
No schooling	2.65	0.91			
Primary level (I–IX)	2.80	0.88			
Secondary level (X–XII)	2.75	0.72			
Tertiary level (≥XIII)	2.66	0.77			
Primary occupation^α^					
a. Service holder	2.78	0.82	5.564	<0.001***	
b. Business	2.86	0.77			b > c*; b > g*; b > i*
c. Student	2.38	0.66			
d. Hawker	2.80	0.91			
e. Labor	2.78	0.89			
f. Farmer	2.77	0.78			
g. Rickshaw puller	2.49	0.88			
h. Auto rickshaw puller	2.92	0.70			h > c*; h > g*; h > i*
i. Others	2.50	0.83			
Religion^ǂ^			1.787	0.074	
Islam	2.72	0.83			
Hindu	2.86	0.73			
Family structure^ǂ^			−4.622	<0.001***	
Single	2.65	0.81			
Joint	2.88	0.82			
Location^α^			154.821	<0.001***	
a. Dhaka City	2.58	0.93			a > b*; a > d*
b. Khulna City	3.51	0.41			b > a* > c* > d*
c. Rangpur City	2.44	0.87			
d. Sylhet City	2.41	0.42			

^ǂ^Indicates t-statistics and ^α^indicates F-statistics (ANOVA); and *p ≤ 0.05; **p ≤ 0.01; ***p ≤ 0.001.

In addition, we employed multivariate linear regression (block-wise enter method) to investigate the factors influencing trust in police among urban men in Bangladesh (Table 4). The model was developed in three stages: Model 1 represented the basic model, while Model 2 included instrumental and expressive models of public confidence in the police and Model 3 controlled for sociodemographic, media influence, satisfaction, and location variables. We conducted thorough checks to ensure that the assumptions of multivariate linear regression were met before finalizing the model. No issues of autocorrelation, multicollinearity, or homoscedasticity were found. The Durbin-Watson test value of 1.55 in the final regression model suggested that autocorrelation was likely not a cause for concern. Homoscedasticity was confirmed for each fitted regression model using residual plots and histograms. Additionally, tolerance values (TV) and Variation Inflation Factor (VIF) were used to assess multicollinearity, and the values in the three models were well below the maximum recommended value of 10, indicating no issue of multicollinearity.

**Table 4:** Multiple regression summary for urban male citizens’ trust in police (*n* = 1,108).

Variables		Model 1				Model 2				Model 3			
		*B*	SE	*β*	Sig.	*B*	SE	*β*	Sig.	*B*	SE	*β*	Sig.
Expressive model	Trust in neighbors	0.378	0.027	0.395	<0.001	0.211	0.024	0.220	<0.001	0.138	0.021	0.144	<0.001
	Law and order situation	0.018	0.046	0.011	0.697	0.063	0.040	0.038	0.116	0.048	0.031	0.029	0.126
Instrumental model	Victimization					0.081	0.051	0.038	0.110	−0.009	0.038	−0.004	0.821
	Concern about personal and family safety					−0.065	0.024	−0.080	0.008	−0.002	0.019	−0.002	0.913
	Perceived crime problem in the area					−0.198	0.026	−0.227	<0.001	−0.091	0.022	−0.105	<0.001
	Personal safety awareness					0.072	0.023	0.076	0.002	0.035	0.018	0.037	0.056
	Neighborhood relative safety					0.260	0.023	0.311	<0.001	0.092	0.019	0.110	<0.001
Socio-demographic	Age (years) (Ref: 19–29)												
	30–39									−0.074	0.050	−0.041	0.138
	40–49									−0.094	0.053	−0.051	0.074
	≥50									−0.201	0.056	−0.099	<0.001
	Marital status: Ever married (Ref: Single)									0.114	0.061	0.049	0.060
	Education (Ref: No schooling)												
	Primary level (I–IX)									−0.018	0.043	−0.010	0.678
	Secondary level (X–XII)									−0.005	0.049	−0.003	0.913
	Tertiary level (≥XIII)									−0.069	0.066	−0.027	0.293
	Primary occupation (Ref: Service holder)												
	Business									−0.074	0.051	−0.041	0.151
	Student									−0.051	0.087	−0.013	0.558
	Hawker									−0.146	0.075	−0.042	0.052
	Labor									−0.136	0.064	−0.054	0.035
	Farmer									−0.038	0.085	−0.010	0.658
	Rickshaw puller									−0.099	0.068	−0.037	0.145
	Auto rickshaw puller									0.001	0.066	0.000	0.994
	Others									−0.085	0.061	−0.033	0.159
	Religion: Islam (Ref: Hindu)									−0.078	0.045	−0.031	0.079
	Family structure: Joint family (Ref: Single)									0.039	0.031	0.023	0.214
Satisfaction with	Governmental law and order operation									0.176	0.022	0.166	<0.001
	Trail of criminal cases by the judiciary									−0.157	0.024	−0.132	<0.001
Media Influence	Believe positive media reports on police									0.253	0.018	0.338	<0.001
	Believe negative media reports on police									0.047	0.013	0.072	<0.001
Location	(Ref: Dhaka City)												
	Khulna City									0.202	0.050	0.106	<0.001
	Rangpur City									−0.228	0.049	−0.110	<0.001
	Sylhet City									−0.250	0.048	−0.136	<0.001
*R* ^2^		0.156				0.411				0.691			
Incremental *R*^2^						0.254***				0.280***			

***p ≤ 0.001; B = b coefficients; SE = standard errors; *β* = beta weights (standardized coefficients).

Model 1 = Expressive model.

Model 2 = Expressive model + Instrumental model.

Model 3 = Expressive model + Instrumental model + Socio-demographic + Satisfaction + Media influence + location.

Furthermore, we conducted principal component analysis (PCA) and reliability analysis to validate and ensure the reliability of the trust in the police scale used in the study, as shown in Table 2. The final model accounted for 69.1% of the variance in the study. In addition to the aforementioned analyses, we conducted principal component analysis (PCA) and reliability analysis to validate and ensure the reliability of the trust in the police scale used in the study, as presented in Table 2. The final model adequately explained 69.1% of the variance in the study.

### 3.5 Ethical consideration

The Institutional Research Board of the Institute of Disaster Management and Vulnerability Studies at the University of Dhaka, Bangladesh (Approval No: ERC (EXT) 10/262022) provided the ethical clearance for this study. Prior to conducting the survey, respondents provided verbal or written consent following ethical guidelines.

## 4 Results

The Principal Component Analysis result showed that the 9-item model developed for this study falls under two major dimensions after evaluating the covariance matrix and Varimax rotation (Table 1). Dimension 1 included six items assessing procedural-based trust, whereas Dimension 2 included three items assessing outcome-based trust on a 6-point Likert scale. The Cronbach’s alpha value of 0.947, as well as KMO and Bartlett’s Test results, denoted that it was a highly consistent single scale to measure trust in police. All nine variables that measured trust in the police were statistically compatible with the final model, which had an eigenvalue of 6.322, explained 70.242% of the variance, and had factor loadings that ranged from 0.757 to 0.871.

Table 2 portrays the descriptive statistics of the dependent variable, showing that male citizens have a moderate level of trust in the police in reference to the scale’s mid-point. The mean score of independent variables depicted that personal safety awareness, trust in neighbors, and neighborhood relative safety had higher mean scores than the scale’s mid-point, while victimization, trust in law-and-order situations, and concern about family safety had lower mean scores. Statistics also showed that the influence of negative reports is higher than positive reports. Satisfaction with government law and order operation, as well as the judiciary, was much lower compared to trust in police. Sociodemographic characteristics are shown in Table 3.

Table 3 depicts the mean scores and differences in the level of trust in police based on respondents’ sociodemographic attributes. Statistics revealed that there was no significant mean difference in trust levels across marital status, education, and religion attributes. In contrast, a significant mean difference was found among the family structure categories (*p* < 0.001), age groups (*p* < 0.001), occupation (*p* < 0.001), and location (*p* < 0.001). The table shows that men belonging to a joint family (*M* = 2.88 ± 0.82) have higher trust than men from a single-family structure (*M* = 2.65 ± 0.81). While younger males aged between 19–29 (*M* = 2.80 ± 0.80) and 30–39 (*M* = 2.80 ± 0.77) years demonstrated higher trust in police, middle-aged (40–49 years old) showed lower trust (*M* = 2.74 ± 0.85) and oldest group (over 50 years old) had the lowest level of trust in police (*M* = 2.56 ± 0.87). In terms of primary occupation, Auto rickshaw puller had the highest mean trust (*M* = 2.92 ± 0.70) followed by business people (*M* = 2.86 ± 0.77) but students had the lowest mean trust (*M* = 2.38 ± 0.66) among all groups. Likewise, regarding location, male participants of Khulna City had the highest mean trust (*M* = 3.51 ± 0.41), and participants of Sylhet City had the lowest mean trust in police (*M* = 2.41 ± 0.42).

In Table 4, a total of three statistical models were developed, and among them, in model 1, researchers explored the impact of expressive model variables on urban male citizens’ trust in Bangladesh police and concluded that only trust in neighbors was statistically significant (*β* = 0.395, *p* ≤ 0.001). In model 2, instrumental model variables were added to expressive model variables, and neighborhood relative safety (*β* = 0.311, *p* ≤ 0.001) had a significant positive association, perceived crime problem in the area (*β* = −0.227, *p* ≤ 0.001) had a negative association and other variables had no association on men respondents’ trust in Bangladesh police. Sociodemographic variables extracted from satisfaction with government operations, media influence, and location variables were integrated into Model 3, which increased the model’s explanatory power from 41.1 to 69.0%. In this final model, Governmental law and order operation (*β* = 0.166, *p* ≤ 0.001), belief in both positive (*β* = 0.338, *p* ≤ 0.001) and negative (*β* = 0.072, *p* ≤ 0.001) media reports on police, and Khulna City residents’ factors (*β* = 0.10, *p* ≤ 0.001) had statistically significant influence on male citizens’ trust in police. On the other hand, belonging to the elderly male (age group ≥50 years) (*β* = −0.099, *p* ≤ 0.01), Trail of criminal cases by the judiciary (*B* = −0.132, *p* ≤ 0.001), Rangpur (*β* = −0.110, *p* ≤ 0.001) and Sylhet (*β* = −0.136, *p* ≤ 0.001) factors negatively influenced the male citizens in trusting the police.

## 5 Discussion

Demographic characteristics and perceptions about police activities have become fundamental factors for evaluating trust within the context of a developing country. Despite the numerous factors correlated with the confidence of the public in the police, this study indicates that urban male citizens in Bangladesh exhibit moderate trust in the police. Although certain studies have suggested that males may have less favorable perceptions of the police for a variety of reasons, such as frequent interactions with police and experiences of being victims of excessive force, the results of this study indicate that urban males still exhibit a moderate level of trust in the police (Taylor et al., 2001; Weitzer and Tuch, 2002; Pryce and Gainey, 2022).

Furthermore, the results highlight the significance of the factor of neighborhood relative safety for urban males, which aligns with the findings of Kääriäinen (2008). Kääriäinen found an inverse relationship between neighborhood insecurity and police trust, indicating that community insecurity diminishes police trust. Concern for family safety and perceptions of crime in the neighborhood often lead to worry among urban males despite relatively stable crime rates in Bangladesh since 2007. The state and its citizens are facing a substantial hazard to their security and safety due to the escalating levels of ethnically, socially, and politically motivated violence (Grant et al., 2022), which might have been reflected in the study’s results by eliciting concerns about family and neighborhood crime.

Fear of crime and family safety are two serious issues in a developing country like Bangladesh, underscoring the need for the police force to improve its image and performance to instill a sense of security and trust in the minds of citizens. While citizens used to believe that the police department was active in fighting crimes in their neighborhoods, they also felt that the police lacked empathy in their interactions with them, sometimes neglecting their complaints, which raises doubts about the sincerity of their service delivery (Ahsan et al., 2024). It should be noted that the ratio of population to police officials in Bangladesh is almost half of the UN standard (Rakib, 2022), leaving each official with a significant workload under various challenging working environments, from political pressure to logistical and skill deficiencies (Grant et al., 2022).

Additionally, despite their vital contribution to Bangladesh’s criminal justice system, the police department is still stigmatized as corrupt (Islam, 2019). The establishment of an anti-terrorism unit within the Bangladesh Police in September 2017, following the Holy Artisan attack in July 2016, marked a notable shift intended to implement a “Zero Tolerance Policy” against terrorism, militancy, and violent extremism (ATU, 2024). Overall, police activities receive widespread coverage in the media, exposing them to broader public perceptions and beliefs. The study’s results demonstrate that media portrayals significantly correlate the perceptions of male citizens, with adverse reports exerting a more significant impact than positive ones. This confirms the presumption about reduced trust in the context of continuous exposure to negative media and police scandals (Callanan and Rosenberger, 2011).

The study also found significant mean differences among the categories of family structure, occupation, and age groups. Belonging to the elderly age group (≥50 years) was discovered to have a negative influence on trust in the Bangladesh police, which is consistent with a similar finding in a study conducted in the USA (Chambers et al., 2020). The male students, exposed to recent social movements where the police’s performance was questioned, may have experienced a decline in confidence compared to occupation group due to interactions with police brutality in Bangladesh. However, overall generational difference showed that younger people had a higher level of trust in police in contrast to the older generation, especially people over 50 years old. A reasonable explanation can be what the older generation experienced over their lifetime, including the several military regimes (1975–1989) and rampant corruption in the early 2000s, where police consistently ranked among the top corrupt institutions in Bangladesh (Siddique, 2014). Additionally, the location was identified as a crucial component in determining trust in the police, with residents of the Khulna division exhibiting a stronger sense of safety and trust in the police compared to other regions, likely influenced by recent socioeconomic development and increased police presence and role in society in that region. In conclusion, the multifaceted factors that influence perceptions and attitudes toward the police force are revealed in this study, which illuminates the complex dynamics of trust in the police among urban male citizens in Bangladesh.

Despite the large sample size, it only reflects the perceptions of the people who had interactions with police in the last 2 years, which can be considered a significant limitation of this study. However, this provided researchers with more reliable data regarding trust in police, which is certain to change following any major event in the future. Moreover, the male-only sample did not present a gender-wise comparison of trust in police, which can be investigated in the future. The inclusion of only two variables from the expressive model also limited the findings of this study, which can be expanded in future research into trust in police.

## 6 Conclusion

In conclusion, following an analysis of the instrumental and expressive models of male citizens’ trust in the police, considering procedural and outcome-based trust, this study revealed a moderate level of trust in the police. However, the level of trust varied significantly across different age groups, family structures, occupations, and locations. Participants expressed optimism regarding recent police initiatives aimed at crime prevention in their communities, particularly operations targeting violent extremism in Bangladesh. Urban males were more concerned for their family’s safety than their own or neighbors’ security. Additionally, male citizens felt that the police lacked empathy, highlighting this as a critical area for future intervention and reform within police departments.

Moreover, police’s negative media portrayal often overshadows their positive activities. To counter this, the police should enhance their social media presence and conduct more effective awareness campaigns, highlighting their successes and the importance of a positive public-police relationship. Public assessments and feedback are crucial indicators of the police’s effectiveness, efficiency, and public trust. However, the police force continues to be the most visible component of the criminal justice system, as it is responsible for a disproportionate amount of social control and crime-related issues. Consequently, the public’s confidence in the police can be substantially increased through enhanced police communication.

## Data Availability

The raw data supporting the conclusions of this article will be made available by the authors without undue reservation.
